# Bridging the gap in dermatology and psychiatry: A scientific rationale

**DOI:** 10.1002/ski2.456

**Published:** 2024-09-12

**Authors:** Isabella J. Tan, Shaunt Mehdikhani, Amy S. Pappert, Paul F. Weber

**Affiliations:** ^1^ Rutgers Robert Wood Johnson Medical School Piscataway New Jersey USA; ^2^ Department of Dermatology Rutgers Robert Wood Johnson Medical School Somerset New Jersey USA; ^3^ Ernest Mario School of Pharmacy Rutgers, the State University of New Jersey Piscataway New Jersey USA

The convergence of dermatology and psychology has given rise to the interdisciplinary field known as psychodermatology. Recently, this field has gained significant recognition and is poised to play a pivotal role in addressing the complex health challenges faced by patients with concurrent dermatological and psychological conditions. In one systematic review evaluating the efficacy of combined psychodermatology clinics, 23 studies yielded data from 1677 patients across 12 countries.^1^ Findings suggest that patients with dermatologic disease and psychosocial comorbidity face barriers to access and gaps in clinician knowledge, with interdisciplinary clinics showing cost reduction and improved outcomes as reported by 87% of studies.^1^ This paper explores the scientific rationale for the driving force of innovation within psychodermatology with a focus on addressing the unmet medical needs in this area and the potential benefits it may offer.

The impetus for positive change in psychodermatology stems from a growing body of literature that underscores the complex relationship between dermatological diagnoses and psychological manifestations.^1^ Numerous studies have revealed the profound impact of psychological factors on dermatological conditions and their treatment outcomes.^1^ It is well‐established that psychological distress can exacerbate dermatological symptoms, contributing to patient distress and diminished treatment efficacy. According to one survey study, many dermatologists have limited awareness of the interdisciplinary nature of psychodermatology, with only 18% reporting a clear understanding of the field and 90% unaware of patient or family resources in their area.^2^

The literature supports the crucial role of dermatologists in recognising and managing psychological manifestations related to dermatological conditions.^3^ Their unique position as frontline medical professionals in this field places them in a prime position to address the interplay between skin health and mental well‐being.

The field of psychodermatology has garnered increasing recognition in recent years. The scientific community, healthcare professionals, and researchers are now faced with a compelling rationale for the need to consider a novel approach and explore its potential in addressing the multifaceted needs of patients afflicted with both dermatological and psychological conditions. This rationale is firmly grounded in a set of scientific principles outlined in Table 1, underscoring the significance of comprehensive care, interdisciplinary collaboration, and innovative solutions. Psychodermatology presents a unique challenge that necessitates a holistic approach.^4^

**TABLE 1:** Scientific principles underpinning psychodermatology.

Scientific principles	Description
Comprehensive care	Psychodermatology demands a comprehensive integrated approach to address the intricate needs of patients. The interconnection of dermatological and psychological aspects of a patient's health underscores the necessity of a broad‐spectrum care model
Interdisciplinary collaboration	The scientific rationale emphasizes the imperative for close collaboration between dermatologists and psychiatrists. This collaboration should not merely be a suggestion but a core element of research and clinical practices within the field of psychodermatology
Innovative solutions	To address the unique challenges of psychodermatology effectively, the scientific community is urged to invest in research and development to formulate and validate innovative solutions. These solutions should be designed to optimize the patient experience, improve access to specialized care, and enhance the overall quality of life for individuals confronting complex dermatological and psychological issues

The introduction of a streamlined referral system can integrate into existing electronic medical records interfaces, presenting a new perspective grounded in scientific principles to address challenges in psychodermatological patient care. This approach may serve to expedite patient care transitions and encourage interdisciplinary collaboration, providing concrete benefits rooted in evidence‐based practices.

By optimising the EMR‐based referral system, delays in referring patients to psychiatric care can be minimized, aligning with the scientific acknowledgement of the pivotal role of timeliness in addressing the multifaceted needs of these patients. This ensures prompt transition of care, thereby enhancing the overall efficiency of patient care. The system may guide referrals based on a patient's position on the psychodermatology spectrum, reflecting a tailored approach. Through facilitating collaboration among dermatologists, psychiatrists, psychologists, and social workers, the system may also support a more holistic and comprehensive strategy.

This approach to integrated psychodermatology has the potential to reform the management of patients dealing with complex cases of superimposed dermatological and psychological conditions. By accelerating specialized psychiatric referrals, this approach can meet the demand for comprehensive care and offer practical advantages for patients and healthcare providers alike (Table 2).

**TABLE 2:** Stakeholders in integrated psychodermatology.

Role	Description
Patients	Individuals over 18 years of age seeking care at dermatology clinics while concurrently dealing with psychological or psychiatric challenges. This patient group requires comprehensive evaluation, diagnosis, and treatment addressing both physical and mental aspects of their health
Dermatologists	Medical professionals at the forefront of diagnosing and treating dermatological conditions. Given the interconnected nature of dermatology and psychology, dermatologists play a pivotal role in identifying psychological manifestations related to skin issues and vice versa
Psychiatrists	Essential providers of specialized mental health expertise and interventions for patients experiencing psychological distress alongside dermatological conditions. Collaboration between psychiatrists and dermatologists is crucial for delivering holistic care
Physician office staff	The support staff within physician offices, including nurses and administrative personnel, play a vital role in facilitating patient care and communication between dermatologists, psychiatrists, and patients

To prioritize patient well‐being, the main objective is to shorten the interval for accessing specialized care, a strategy with numerous benefits. First, this aligns with improved clinical success, as prompt access to psychiatric assistance alongside dermatological treatment may enhance outcomes. Furthermore, reducing the delay to treatment can alleviate psychological distress, which is especially crucial for patients coping with the emotional challenges of their conditions.

For physicians, the focus lies on establishing an efficient referral workflow, aimed at simplifying processes for medical professionals. This initiative offers several key benefits, starting with streamlining the referral process, thereby alleviating the administrative burden on physicians and their staff (Table 2). Consequently, referrals are processed efficiently, evolving the system into a validated means of patient referral and enhancing the reliability of patient care. Moreover, this approach facilitates smoother transitions for physicians, allowing for the allocation of more time to direct patient care and less to administrative tasks.

In one case series, the integrated service of a dermatologist and a liaison psychiatrist demonstrated significant benefits in patient outcomes and cost savings.^5^ This approach achieved an average cost saving of £2038.33 per patient (approximately $2539.05 USD).^5^ This model also effectively addressed complex presentations, with 88% of patients requiring psychiatric medication.^5^ By merging expertise from both disciplines, the importance of integrated care in improving patient well‐being and reducing healthcare cost was highlighted.

This integrated care model aligns with calls for expanded psychodermatology services, emphasizing its value in meeting the multidisciplinary and holistic needs of patients while maximizing healthcare resources. It is founded on the principle that timely specialized care is crucial for addressing the intersection of dermatological and psychological conditions, offering practical benefits rooted in scientific understanding and enhancing patient outcomes.

The robust rationale supporting the proposed psychodermatology viewpoint warrants widespread acceptance within the scientific community. Collaborative efforts among dermatologists, psychiatrists, and researchers are essential for investigating the long‐term efficacy of this approach and its impact on patient outcomes. Through integrating scientific research and evidence‐based practices, the gap between dermatology and psychiatry can be bridged, offering renewed hope and healing to patients and physicians. Active engagement in this journey has the potential to propel the field of psychodermatology forward, enhancing the quality of life for individuals facing complex health challenges. Collaboration, as it relates to psychodermatology, results in better patient care, manifesting in more timely interventions, comprehensive treatments, heightened patient satisfaction and relief of distress, all at a reduced cost. It is imperative for the scientific and clinical communities to recognize and proactively support the potential of this innovative approach to advance patient care and well‐being and healthcare efficacy.

## CONFLICT OF INTEREST STATEMENT

The authors declare no conflicts of interest.

## AUTHOR CONTRIBUTIONS

**Isabella J. Tan**: Conceptualization (equal); data curation (equal); formal analysis (equal); investigation (equal); methodology (equal); project administration (lead); visualization (equal); writing—original draft (lead). **Shaunt Mehdikhani**: Conceptualization (equal); data curation (equal); formal analysis (equal); investigation (equal); methodology (equal); project administration (equal); visualization (equal); writing—original draft (equal). **Amy S. Pappert**: Supervision (lead); writing—review & editing (lead). **Paul F. Weber**: Supervision (lead); writing—review & editing (lead).

## ETHICS STATEMENT

Not applicable.

## PATIENT CONSENT

Not applicable.

## Data Availability

The data underlying this article will be shared on reasonable request to the corresponding author.
